# The coupling coordination between digital village construction and rural healthcare service efficiency in China: dynamic evolution, spatial difference and driving factors

**DOI:** 10.3389/fpubh.2025.1669695

**Published:** 2025-10-13

**Authors:** Kaizheng Wang, Baoyang Ding, Xiaoyu Huang, Hui Pang, Jun Hu

**Affiliations:** School of Health Management, Shandong University of Traditional Chinese Medicine, Shandong, China

**Keywords:** digital village, healthcare services, coupling coordination, kernel density estimation, spatial Markov chain

## Abstract

**Background:**

Promoting coordination between digital village construction and rural healthcare service efficiency is central to advancing rural social progress and implementing the Healthy China strategy, and has drawn growing attention in public administration and health economics.

**Methods:**

Using panel data from 29 provinces across China spanning 2015–2022, the study employed the coupling coordination degree model to assess the level of coupling coordination degree (CCD) between digital village construction and the effectiveness of rural healthcare services. The Dagum’s Gini coefficient was applied to analyze regional disparities, while kernel density estimation and spatial Markov chain were utilized to examine reveal their spatio-temporal dynamic evolution patterns. Employed quantile regression to analyze the primary factors influencing the CCD.

**Results:**

The national coordination level has steadily improved from 0.589 to 0.665, but some provinces have yet to reach the coordination stage. The national Gini coefficient has risen from 0.148 to 0.158, with within-regional disparities continuing to increase, while inter-regional disparities have gradually narrowed. Kernel density analysis showed that the right tail of the eastern region continued to widen, with benchmark provinces emerging within the region, while the central and western regions showed a trend towards evolution of double peak and multi-peak, respectively, with increased differentiation within the region. Spatial Markov chain results indicate that stable synergistic relationships exist between high-level regions or low-level regions; medium-to-high-level regions exhibit a certain degree of siphoning effect on surrounding medium-to-low-level regions. Economic, government support, and health needs are positive factors driving the CCD development, while health human capital and urbanization rate are negative factors.

**Conclusion:**

The coordinated development of digital village construction and rural healthcare service efficiency has achieved positive results, but issues such as regional development imbalances, intensified within regional differentiation trends, development lock-in, and resource siphoning still require attention. The CCD is influenced to varying degrees by social, economic, and demographic factors. In the future, efforts should be made in two areas: regional balance and interregional coordination, and strengthening government support to promote the effective allocation of resources and the coordinated advancement of digital healthcare.

## 1 Introduction

Since the State Council of the People’s Republic of China successively issued the “National Health System Development Plan (2015–2020)” and the “Opinion of the State Council on Actively Promoting the Integration of Internet Plus in Medical and Health Care” in 2015, digitalization has been formally incorporated into the strategic blueprint for the development of medical and health care, marking the entry of the transformation of medical service models into a new phase (1, 2). With the swift advancement and broad utilization of digital technology are effectively empowering the modernisation of China’s healthcare services, leading to significant changes in public healthcare services and management methods. Compared to urban areas, rural regions in China have relatively weak digital infrastructure, with lagging network infrastructure and information and communication technology support, which has to some extent hindered the widespread adoption of digital healthcare services in rural areas. To address this, since the initial “Digital Village Strategy” proposal in 2018 (3), China has continued to promote the development of digitalisation in rural areas. In 2022, the government issued the “Digital Village Development Action Plan (2022–2025),” proposing to use digital technology to improve the quality of life in rural areas and focus on bridging the digital divide between urban and rural areas (4). Meanwhile, China’s current rural healthcare system still faces prominent issues such as low service efficiency, imbalanced resource allocation, and significant gaps in urban–rural healthcare services (5), and the traditional resource allocation model is no longer sufficient to meet the growing health needs of rural residents. However, as digital village initiatives continue to expand, the merging of technological advancements with rural medical services has emerged as a key strategy for enhancing healthcare standards in the countryside and bridging the health disparity between urban and rural populations (6). Therefore, in 2024, the “Digital Village Development Guidelines 2.0” mentioned the need to continue promoting the development of digital health in rural areas and extend digital medical technology to primary healthcare institutions such as township health centres and village clinics (7). Against the backdrop of the comprehensive advancement of rural revitalisation, the transformation brought by digital technology also offers new possibilities for rural healthcare development. Therefore, building a healthy rural community centred on digital construction and healthcare development is expected to become an important driving force for realising the Healthy China strategy and the goals of comprehensive rural revitalisation.

The innovation of this study is mainly reflected in the following three aspects. First, by calculating the coupling coordination degree (CCD) between digital village construction and rural healthcare service efficiency, this study fills the gap in existing research on this topic from a rural perspective. Second, this study proposes and constructs a two-way interactive mechanism between the two, enriching the theoretical framework for the coordinated development of digitalization and medical services. Third, addressing the common shortcomings of existing studies, which have primarily focused on measurement while neglecting spatial effects and unclear influencing factors, this study combines empirical models with spatial data analysis. It not only reveals the regional differences and spatio-temporal evolution characteristics of the coordinated development between digital village construction and rural healthcare services but also identifies their key influencing factors, thereby providing data support and theoretical basis for policy optimization.

## 2 Literature review

There is relatively little existing research on the CCD of digital village construction and rural healthcare services. Related studies are mostly concentrated on the relationship between the digital economy and medical services, which can be broadly divided into three categories.

The first category of research emphasises the role of the digital economy in promoting healthcare services. Several scholars contend that advances in digital infrastructure and the broader adoption of digital tools have greatly enhanced access to medical resources and boosted the overall efficiency of health services (8–10). As research has progressed, some researchers have found that the spatial spillover effects of digital development have significantly increased. Through the intervention of technology and capital, the level of public health services in surrounding areas has been improved (11). In addition, some scholars have found that the digital economy promotes information flow and talent mobility, which helps to optimise resource allocation (12, 13). At the same time, existing research has found that digital tools have shown high value in public health emergencies, improving the efficiency and capacity of health units (14, 15), and tools such as telemedicine and online pharmacies have improved the accessibility and equity of healthcare in remote areas (16, 17). However, some studies have pointed out that without policy support and infrastructure, digitisation may exacerbate service inequalities between regions and widen the gap in the quality of public services (18, 19).

The second category of research focuses on the role of healthcare service development in promoting the digital economy. Some scholars have argued that the digital transformation of healthcare services has not only stimulated the development of digital markets and information infrastructure, but also accelerated medical technology innovation and promoted the optimisation of the digital economy system (20). On the one hand, the digital transformation of healthcare services provides application scenarios and demand for digital infrastructure, becoming an important pillar of the digital economy (12). Simultaneously, evidence from the U.S. suggests that digital health interventions for patients have somewhat enhanced their self-management skills (21). Residents can use digital tools such as mobile health apps (22) to access health information in a timely manner, thereby improving their health literacy and disease prevention awareness (23, 24), thereby reducing the burden of disease in rural areas and improving the overall efficiency of medical services. On the other hand, the intelligent upgrading of basic public health services has facilitated the rise of Internet hospitals and other models, gradually realising the digitisation of the whole process of healthcare services and helping the expansion of new business models in the digital economy (25, 26).

The third category of research focuses on measuring CCD between the digital economy and health services. For example, Liu et al. used a coupling coordination degree model to measure the level of coordinated development between the digital economy and healthcare services, finding that CCD showed an overall upward trend, but the growth rates varied significantly (27). In addition, researchers have calculated CCD for China’s digital rural development and rural healthcare services, finding that CCD for the former is higher than that for the latter in eastern China compared to central and western China (28). Some scholars have also calculated the CCD for the digital economy and basic health services and found that it exhibited obvious spatial clustering characteristics (29). Chen et al. used the Tobit model to analyse the factors influencing the CCD between China’s digital economy and public health services, finding that the level of economic development is the primary driving force behind improving coordination (30).

Summarizing previous research, studies tend to focus on the unilateral relationship between digital infrastructure development and healthcare services. Only a few scholars have conducted research on the bidirectional interaction between the two, but there are still gaps in terms of temporal and spatial analysis. Secondly, existing research has primarily focused on urban areas, overlooking the integration of digital infrastructure and healthcare systems in rural areas. Due to significant differences between urban and rural areas in terms of industrial structure, functional positioning, resource endowment, and main characteristics, the construction of digital villages is not a simple extension of the smart city model (31). Therefore, against the backdrop of widening gaps in urban and rural healthcare, how should China and other developing countries effectively prioritise the coordinated development of digitalisation and healthcare services in rural areas?

To address these research gaps, this study calculated the digital rural construction level and rural healthcare service efficiency of 29 Chinese provinces from 2015 to 2022, and to measure their CCD. This study aims to explore regional differences, spatio-temporal dynamic trends and potential influencing factors of CCD between the two systems, and finally to propose policy recommendations.

## 3 Theoretical mechanism

**Figure 1:**
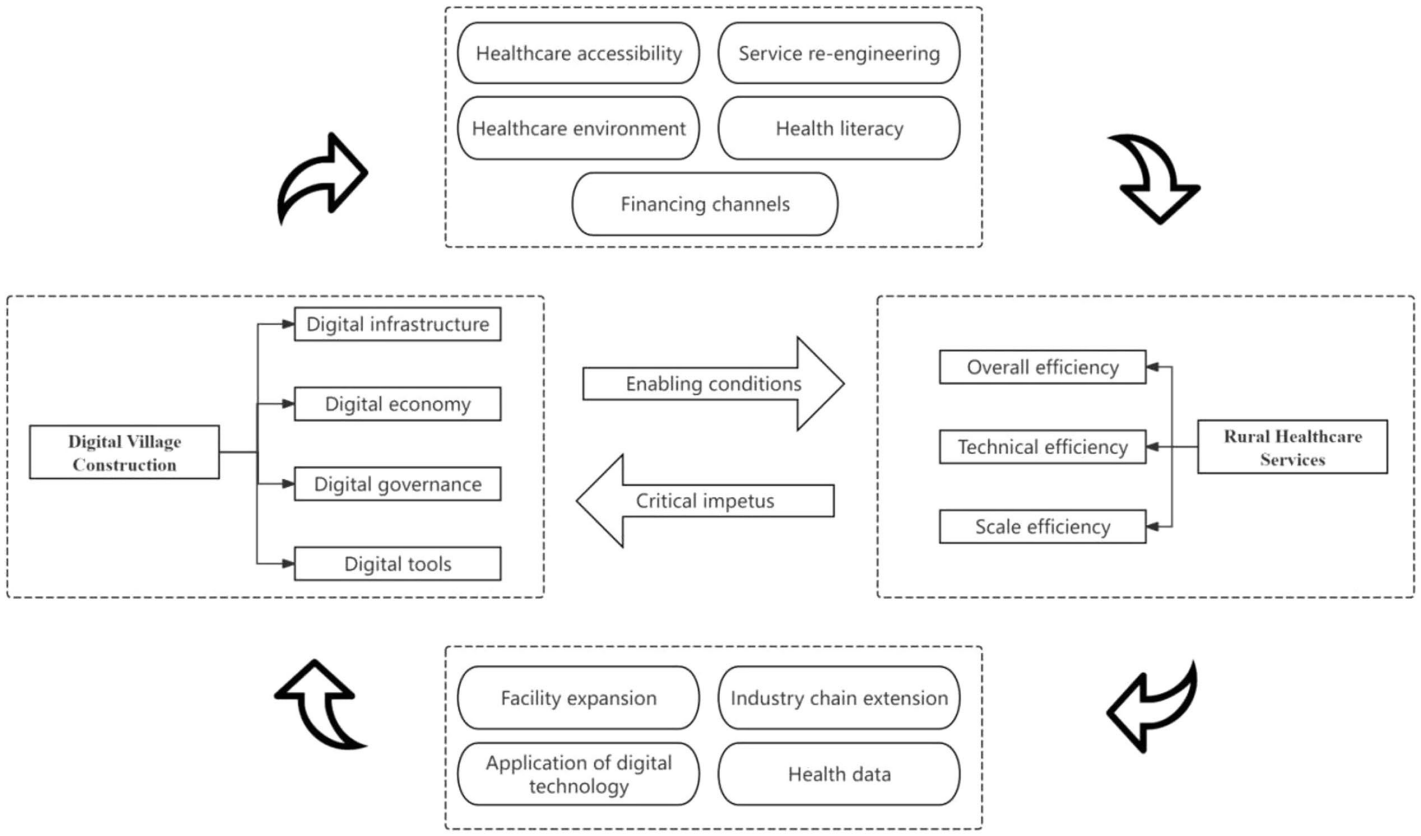
Theoretical framework of interaction between digital village construction and rural healthcare service.

The theory of coupling coordination revolves around two fundamental principles: interconnectedness and synergy. “Coupling” describes the reciprocal relationship and reliance between systems, where multiple entities dynamically interact and shape one another (32). “Coordination” focuses on exchanging matter, information, and energy across systems to foster mutual benefit and sustainable growth (33). This study explores the interactive relationship between digital rural construction and the efficiency of rural healthcare services, viewing them as two systems operating in synergy and mutually reinforcing each other through a dynamic mechanism. The theoretical framework for the interaction between digital rural development and rural healthcare services is shown in Figure 1.

### 3.1 Digital villages construction can effectively promote the development of rural healthcare services

Digital villages construction provides systematic enabling conditions for improving the efficiency of rural healthcare services through the synergistic effects of infrastructure, economic development, governance systems, and digital tools. First, the improvement of digital infrastructure has broken the time and space constraints of medical services and enhanced the accessibility of primary medical services (34). At the same time, digitization has promoted the transformation of traditional medical service processes, such as electronic health records and medical test results, which can be shared between different institutions, thereby supporting the efficient operation of services such as remote consultation and two-way referrals (35). Second, the development of the digital economy has expanded payment and financing channels. On the one hand, it has made medical insurance settlement and drug supply chain management more efficient, significantly reducing transaction costs for residents receiving medical services (36). On the other hand, it has enhanced the financing capacity of rural medical and health institutions by increasing diversified sources of investment, thereby ensuring the efficient operation of the rural medical system. Furthermore, digital governance has improved government administrative efficiency and regulatory enforcement by establishing a unified coding system, interface standards, and privacy and security regulations. This has helped to establish sound institutional safeguards and create a favorable environment for the high-quality development of rural healthcare services (37). Additionally, the application of digital tools such as digital health education platforms has promoted the improvement of health literacy among rural residents, enhanced disease prevention awareness and self-health management capabilities, and helped optimize the quality of disease diagnosis and follow-up (23). These mechanisms work together to improve the technical efficiency of healthcare services in terms of resource input and output, enhance economies of scale through regional resource sharing, and drive overall efficiency upward.

### 3.2 Rural healthcare services contribute to the high-quality development of digital villages

Improvements in the efficiency of rural health services have also become a key driver for the further development of digital villages. On the one hand, the digitization of rural health services has created a strong demand for a digital environment with higher bandwidth and more stable networks, thereby driving the continuous expansion of digital infrastructure (38, 39). On the other hand, as healthcare services become more efficient, the extension of the healthcare service chain has promoted the rapid development of related digital industries such as equipment, cold chain, and e-commerce for medical devices and supplies. For example, wearable health devices are gradually entering the village-level market, and data-driven drug supply chains, disease warning services, and smart healthcare services can be piloted in rural areas. In addition, rural residents’ actual experience with digital healthcare services has increased their acceptance of digital technologies, prompting them to explore a wider range of digital technology services, which in turn provides more application scenarios for digital technologies (40). At the same time, village health clinics and township health centers serve as the frontline for health data collection and transmission. The large amount of health data generated by their medical activities provides an accurate decision-making basis for rural governance, helping to optimize the management of key populations and the allocation of health resources according to demand (41). Furthermore, through the county-level medical consortium collaboration platform, these data have further enhanced the level of cooperation and coordination in surrounding areas.

## 4 Materials and methods

### 4.1 Data source

This study focuses on 29 provinces (cities and autonomous regions) in China (excluding Hong Kong, Macao, and Taiwan), covering the period from 2015 to 2022. Due to the lack of data on rural healthcare services in Beijing and Shanghai, we have only selected data from the 29 provinces in mainland China. This is the same as what other research institutes have done (42). The data for this study primarily come from the “China Statistical Yearbook,” “the China Rural Statistical Yearbook,” “provincial statistical yearbooks,” “the China Health Statistical Yearbook,” “the China Health and Family Planning Statistical Yearbook,” and “the Digital Finance Research Institute of Peking University.” According to the classification standards of the National Health Commission, China’s 29 provinces are divided into three regions: East, Central, and West (43).

### 4.2 Research methods

This study employs the CRITIC method to calculate the weights of indicators for digital rural development and calculate the score. Secondly, the super-efficiency slack-based measure (SBM) model is used to calculate the efficiency scores of rural healthcare services. After calculating the scores of the two systems, the CCD between them is calculated using the coupling coordination degree model, and the regional differences in their CCD are measured using the Gini coefficient. Kernel density analysis is used to depict the evolution trends of CCD over time, and spatial Markov chains are used to depict the evolution trends of CCD in space.

#### 4.2.1 CRITIC weight method

CRITIC weighting approach is an objective methodology for assigning weights to evaluation criteria. It assesses both the relative importance of each indicator and the degree of conflict or overlap between them, ultimately producing a balanced, data-driven weighting system for measurement factors and offers greater benefits compared to the entropy and standard deviation methods (44). To standardize the indicators and remove the effects of differing measurement units, the range method was applied to normalize each data set (45). The formula is as shown in Equations 1–5:

(1) $${\bar{x}}_{j}=\frac{1}{n}\sum_{i=1}^{n}x_{\mathrm{ij}}$$

(2) $$S_{j}=\sqrt{\frac{\sum_{i=1}^{n}{\left(x_{\mathrm{ij}}-{\bar{x}}_{j}\right)}^{2}}{n-1}}$$

(3) $$R_{j}=\sum_{i=1}^{p}(1-r_{\mathrm{ij}})$$

(4) $$C_{j}=S_{j}(1-r_{\mathrm{ij}})=S_{j}\times R_{j}$$

(5) $$W_{j}=\frac{C_{j}}{\sum_{j=1}^{p}C_{j}}$$

Where $S_{\mathrm{ij}}$ represents the standard deviation of indicator j, $r_{\mathrm{ij}}$ represents coefficient between evaluation index i and j, $C_{j}$ represents the amount of information of j, $W_{i}$ represents the objective weight of indicator j.

To accurately reflect the level of digital village development, this study draws upon established research (28, 46–49) and develops a comprehensive evaluation framework based on accessible data. The assessment system breaks down digital village progress into four key dimensions: digital infrastructure, digital life, digital services, and digital economy. The indicators are shown in Table 1.

**Table 1:** Indicator system for digital village construction.

Target layer	Index layer	Unit	Weight
Infrastructure digitization	The number of computers per 100 households in rural areas (24)	100 households	0.082
	The number of smartphones per 100 households in rural areas (40)	100 households	0.038
	The number of televisions per 100 households in rural areas (24)	100 households	0.090
	The number of subscribers with broadband access per 10,000 rural households (40)	10,000 households	0.086
	The total distance covered by rural postal delivery routes (24)	Kilometers	0.096
Life digitization	Consumption expenditure on transport and communication per rural household (24)	Yuan per person	0.089
	The average amount of rural electricity consumption per 10,000 people (43)	Billion kilowatt-hours	0.064
Service digitization	The amount of total rural village committees (24)	Pieces	0.098
	The number of village medical institutions (24)	Pieces	0.143
	The amount spent on healthcare per person in a rural area (44)	Yuan per person	0.094
Economic digitization	Digital inclusive finance development index (40)	—	0.119

#### 4.2.2 Super efficiency SBM model

The traditional data envelopment analysis (DEA) model has been widely applied in studies measuring the efficiency of healthcare services (50). However, this model uses radial and angular measurements, which cannot address the slack effects caused by inputs and outputs, nor can it further compare Decision-Making Units (DMUs) with efficiency values equal to the maximum value of “1.” To address these issues, Tone and Xue proposed the SBM model based on non-radial slack (51) and the super-efficiency DEA model (52), respectively. The super-efficiency SBM model combines the two models and offers superior advantages over the traditional DEA model (53). Therefore, this study uses the super-efficiency SBM model to calculate the efficiency of rural healthcare services, with the formula as shown in Equation 6:


(6) $$\mathrm{TE}=\frac{1+\frac{1}{m}\sum_{i=1}^{m}\frac{w_{i}^{-}}{x_{\mathrm{ik}}}}{1-\frac{1}{s}\sum_{r=1}^{s}\frac{w_{r}^{+}}{y_{\mathrm{rk}}}}$$

Where TE represents the rural healthcare service efficiency; $x_{\mathrm{ik}}$ and $y_{\mathrm{rk}}$ represent the i-th input and r-th output in region k, respectively; $w_{i}^{-}$ and $w_{r}^{+}$ represent the slack variables of inputs and outputs, respectively.

Based on existing research on medical service efficiency (54, 55), this paper divides the efficiency of rural healthcare services into two parts: input indicators and output indicators. Input indicators include the number of township health centers, the number of village health clinics, the number of rural health technicians, and the number of beds. Output indicators include the number of rural primary care visits and the number of hospital discharges. Specific indicators are shown in Table 2.

**Table 2:** Indicator system for the efficiency of rural healthcare services.

Target layer	Index layer	Unit
Inputs	The number of township health centers	Pieces
	The number of village clinics	Pieces
	The number of rural health technicians	Person
	The number of beds in village medical institutions	Pieces
Outputs	The number of diagnosis and treatment visits from village medical institutions	Person
	The number of discharges from village medical institutions	Person

#### 4.2.3 Coupling coordination degree model

The coordination coupling model serves as a tool for assessing and examining the synergistic growth among multiple interconnected systems, capturing both their mutual reliance and inherent limitations (56). Therefore, this study utilises the coupling coordination degree model to calculate the CCD between digital village construction and rural healthcare service efficiency. The formula for calculating the CCD is as shown in Equations 7–9:

(7) $$C={\left[\frac{U_{1}U_{2}}{{\left[\frac{U_{1}+U_{2}}{2}\right]}^{2}}\right]}^{\frac{1}{2}}$$

(8) $$D=\sqrt{C\cdot N}$$

(9) $$N=\alpha U_{1}+\beta U_{2}$$

Where C represents the coupling degree. D represents the CCD. The higher values indicate stronger links correlation of the two systems. $U_{1}$ and $U_{2}$ are the comprehensive evaluation indicators for rural village construction and the efficiency of rural healthcare services, respectively. N is the comprehensive evaluation index for the overall coordination of the two systems. α and β denote the respective contribution coefficients of the two systems. Given that digital villages and rural healthcare services form a symmetrical relationship within the model, both are indispensable. Arbitrarily setting different α and β values would introduce subjective bias. Therefore, referencing relevant literature (29), it is assumed that the contributions of the two systems to the coordinated development of coupling are equal, α=β=0.5. Furthermore, we categorise CCD values into three interpretable tiers: 0–0.4 (Antagonistic period), 0.4–0.6 (Break-in period), and 0.6–1.0 (Coordination period). The grading of the CCD level of the two systems is shown in Table 3.

**Table 3:** Hierarchy of CCD between digital village development and rural healthcare service efficiency.

Coordination level	CCD value	Coupling development type
Antagonistic period	[0.0–0.1]	Extreme dysregulation and decline
	(0.1–0.2]	Severe dysregulation and decline
	(0.2–0.3]	Moderate dysfunctional decline
	(0.3–0.4]	Minor disorder
Break-in period	(0.4–0.5]	Proximity coordination
	(0.5–0.6]	Barely coordinated
Coordination period	(0.6–0.7]	Primary coordination
	(0.7–0.8]	Intermediate coordination
	(0.8–0.9]	Well-coordination
	(0.9–1.0]	High-quality coordination

#### 4.2.4 Dagum’s Gini coefficient and its decomposition

Dagum’s Gini coefficient can examine spatial inequality issues by decomposing them into subgroups, allowing for a more detailed division of overall inequality into intra-regional inequality, inter-regional inequality, and overlapping inter-regional inequality. Therefore, this study uses Dagum’s Gini coefficient to examine regional heterogeneity of the CCD between the two systems. The formula is as shown in Equation 10:

(10) $$G=\sum_{i=1}^{k}\sum_{h=1}^{k}\sum_{j=1}^{n_{j}}\sum_{r=1}^{n_{h}}|y_{\mathrm{ij}}-y_{\mathrm{hr}}|/2n^{2}\bar{y}$$

Where n represents the number of provinces, and k represents the number of regions. $n_{i}$ and $n_{h}$ represent the number of provinces in region i and region h, respectively. $y_{\mathrm{ij}}$ (or$\;y_{\mathrm{hr}}$) represents the CCD of province j (or r) in region i (or h), and is the average CCD.

#### 4.2.5 Kernel density estimation

This study uses kernel density curves to describe the temporal evolution of the CCD, and estimates the probability density. Assuming it describes random variable x’s density., the formula is as shown in Equation 11:

(11) $$f(x)=\frac{1}{N_{h}}\sum_{i=1}^{n}K(\frac{X_{i}-x}{h})$$

Where N is the number of observations, $X_{i}$ is the independent distribution of observations, x is the mean value, K is the kernel density function, and h is the bandwidth. The article selects the dynamic distribution level of the CCD between the two systems estimated by a high-precision Gaussian kernel function, whose functional expression is as shown in Equation 12:

(12) $$K(x)=\frac{1}{\sqrt{2\pi }}e^{\left(-\frac{x^{2}}{2}\right)}$$

#### 4.2.6 Spatial Markov chain

By constructing a Markov transition probability matrix, we analysed the probability of transition between different states of CCD and explored the spatial dynamic evolution trend of it over time. We discretised CCD into k state types and calculated the changes and probabilities of each state (57), thereby approximating the evolutionary process of the CCD as a Markov process. The formula is as shown in Equations 13, 14:

(13) $$\begin{array}{l} P\{X_{t}=j|X_{t-1}=i_{t-1}|X_{t-2}=i_{t-2}|,\ldots ,|X_{0}=i_{0}\}\\ =P\{X_{n}=j|X_{n-1}\}=P_{\mathrm{ij}} \end{array}$$

(14) $$P_{\mathrm{ij}}=\frac{n_{\mathrm{ij}}}{n_{i}}$$

In this study, we employed a quartile classification method to categorise CCD into four distinct levels: low (k = I), medium-low (k = II), medium-high (k = III), and high(k = IV). An adjacency matrix was employed to incorporate spatial lag effects into the traditional Markov chain transition probability matrix, converting it into a conditional transition probability matrix to account for spatial spillover effects from geographic proximity on CCD evolution, thereby analysing the dynamic evolution trend of the CCD between digital villages and rural healthcare service efficiency under the influence of neighbouring cities.

#### 4.2.7 Quantile regression method

As an advanced econometric method, quantile regression can deeply analyse the mechanism by which explanatory variables influence the conditional distribution of the dependent variable at different quantiles. Unlike ordinary least squares regression (OLS), which can only estimate the conditional mean function, this method constructs a conditional quantile function to comprehensively reveal the heterogeneous characteristics of the relationship between variables (58). In this study, empirical analysis was conducted using five quantiles from 0.1 to 0.9 to explore the differentiated effects of various influencing factors on the coordinated development level of digital rural construction and rural healthcare service efficiency in China. The formula is as shown in Equation 15:


(15) Qyi(δXi)=Xi′+β(δ)+ε(δ)

Qyi(δXi) represents the quantile of the δth condition of $y_{i}$, and β(δ) represents the estimation of the δth quantile coefficient, ε(δ) represents the error term.

In order to further analyse the factors that may affect the construction of digital villages and the efficiency of rural health services, this study refers to existing research (29, 30, 59–61) and combines data availability to analyse the factors that may affect the CCD of the two systems, mainly from the perspectives of economic development level, government institutional support, and population health needs. Finally, factors such as economic development level (LED), government support (GOV), rural residents’ health needs (HDI), digital innovation capacity (DIN), health human capital (HHC), and urbanisation rate (URB) were selected as explanatory variables. Using CCD data from 29 provinces across China from 2015 to 2022 as the explanatory variable. The specific definitions of the explanatory variables are shown in Table 4.

**Table 4:** Factors affecting the CCD between two systems.

Types	Variable	Variable symbol	Variable description	Unit
Explained variable	Coupling coordination degree	D	Calculation results of the coupling coordination model between two systems	
Explanatory variable	Economic development level	LED	Per capita GDP	Yuan
	Health needs of rural residents	HNL	Per capita healthcare expenditure of rural residents	Yuan/person
	Digital innovation capabilities	DIN	Share of R & D spending to GDP	%
	Government support	GOV	Government fiscal expenditure on healthcare	Yuan
	Health human capital	HHC	Number of health technicians per 1,000 people in rural areas	person
	Urbanisation rate	URB	Urbanisation rate = year-end urban population / total population	%

## 5 Results

### 5.1 Evaluation of the CCD between digital village construction and rural healthcare service efficiency

From the perspective of digital village construction levels, China’s digital village development level has seen steady progress between 2015 and 2022, with nationwide scores climbing from 0.238 to 0.377 on average. The eastern provinces maintained their frontrunner position throughout this period, while the western regions continued to lag behind. In contrast, the efficiency of rural healthcare services first increased and then decreased: the national average peaked at 0.716 in 2016, then dropped to 0.570 in 2021, and recovered to 0.650 in 2022; Rural healthcare service efficiency in the eastern region has remained consistently high, but it is a different story in the central and western. Back in 2015, the efficiency of healthcare services in central and western regions was 0.689 and 0.633, respectively. Fast forward to 2022, and the efficiency of healthcare services in central and western regions had fallen to 0.611 and 0.560, respectively., indicating limited improvement in rural healthcare service efficiency. The overall CCD of the two systems remains in the primary coordination phase, with the national average increasing from 0.598 to 0.665. However, it is worth noting that there was a decline between 2019 and 2021, which may be attributed to the impact of the COVID-19 pandemic, leading to a decline in rural healthcare service efficiency and obstacles in the progress of digital village construction, thereby weakening the synergistic development mechanism between the two systems and causing a temporary decline in the CCD. From 2015 to 2022, the eastern region’s average CCD was 0.727, indicating that the eastern region has long been in an intermediate coordinated stage. In contrast, the central region’s average CCD was 0.654, exceeding the western region’s 0.574 from 2015 to 2022. Overall, China’s digital rural construction and rural healthcare efficiency both exhibit a distribution pattern that is stronger in the east than in the west. The results of the CCD are presented in Table 5.

**Table 5:** Level of digital village construction, level of rural healthcare service efficiency, and the CCD from 2015 to 2022.

Year	Level of digital village construction				Level of rural healthcare service efficiency				Level of CCD			
	East	Central	West	National	East	Central	West	National	East	Central	West	National
2015	0.319	0.257	0.166	0.238	0.839	0.689	0.633	0.712	0.692	0.622	0.512	0.598
2016	0.362	0.287	0.200	0.274	0.830	0.705	0.638	0.716	0.720	0.649	0.557	0.633
2017	0.419	0.332	0.247	0.324	0.827	0.708	0.619	0.708	0.754	0.680	0.596	0.668
2018	0.392	0.330	0.235	0.310	0.819	0.700	0.588	0.690	0.736	0.675	0.576	0.653
2019	0.431	0.357	0.259	0.339	0.754	0.625	0.582	0.647	0.736	0.669	0.587	0.656
2020	0.430	0.361	0.270	0.345	0.715	0.565	0.540	0.601	0.715	0.632	0.580	0.637
2021	0.460	0.386	0.297	0.372	0.678	0.529	0.517	0.570	0.708	0.639	0.582	0.636
2022	0.457	0.394	0.306	0.377	0.805	0.611	0.560	0.650	0.752	0.664	0.600	0.665
Mean	0.409	0.338	0.247	0.322	0.783	0.642	0.585	0.662	0.727	0.654	0.574	0.643

**Figure 2:**
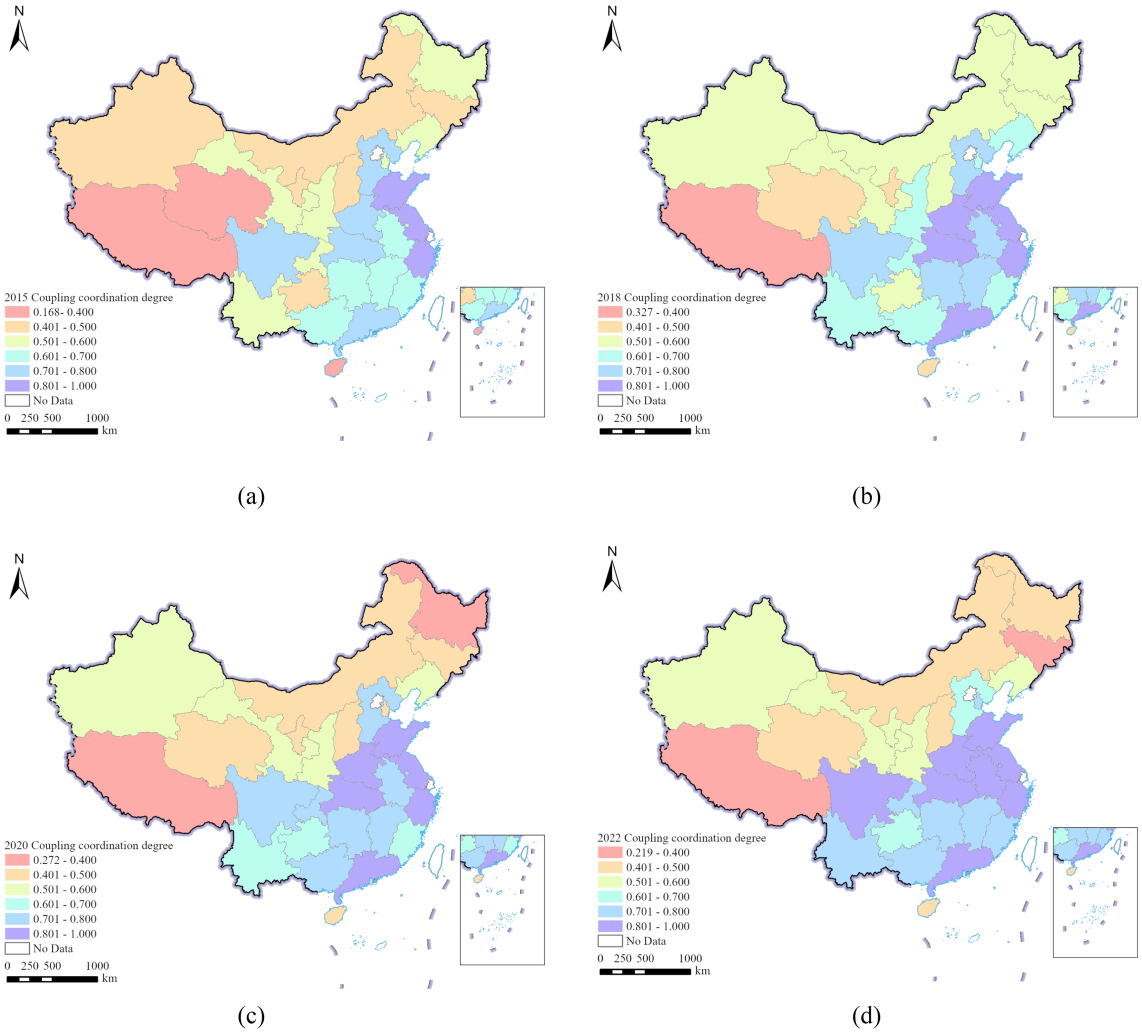
Spatial distribution of CCD in 29 Chinese provinces, **(a)** 2015, **(b)** 2018, **(c)** 2020, **(d)** 2022.

From a spatial distribution perspective, only Sichuan Province in the western region has maintained a high level of CCD in the coordinated stage. Chongqing Municipality, Yunnan Province, and Guizhou Province have transitioned from the transitional coordination stage to the coordinated stage, with an increase in CCD, while the Tibet Autonomous Region remains in the uncoordinated stage. In the central region, Henan Province, Hubei Province, Hunan Province, Anhui Province, and Jiangxi Province have remained in the coordinated stage, with an increase in CCD. However, Jilin Province has transitioned from the transitional coordination stage to the uncoordinated stage, with a decrease in CCD; In the eastern region, Shandong Province, Jiangsu Province, Zhejiang Province and Guangdong Province remain a high level of CCD in the coordination phase. Hainan Province has transitioned from the uncoordinated stage to the transitional coordination phase, with an increase in the level of CCD. The results are shown in Figure 2.

### 5.2 Regional heterogeneity analysis of the CCD between two systems

The CCD between China’s digital village construction and rural healthcare service efficiency exhibits a distinct spatial differentiation pattern. To further analyse the sources of this spatial differentiation pattern, this study uses the Gini coefficient decomposition method to examine the regional heterogeneity of the CCD between two systems. The result is shown in Table 6.

**Table 6:** Gini coefficient and decomposition of the CCD between two systems from 2015 to 2022.

Year	Gini coefficient	Within-region Gini coefficient			Inter-region Gini coefficient			Contribution rate		
		East	Central	West	East–Central	Central–West	East–West	Within-region	Inter-region	Hypervariable Density
2015	0.148	0.123	0.097	0.147	0.128	0.147	0.190	0.290	0.473	0.237
2016	0.132	0.116	0.091	0.123	0.122	0.128	0.167	0.290	0.449	0.261
2017	0.119	0.105	0.083	0.108	0.113	0.114	0.150	0.289	0.456	0.254
2018	0.127	0.115	0.093	0.115	0.118	0.126	0.158	0.292	0.444	0.264
2019	0.135	0.128	0.104	0.128	0.129	0.129	0.162	0.306	0.386	0.308
2020	0.16	0.151	0.158	0.133	0.168	0.16	0.176	0.306	0.297	0.397
2021	0.165	0.165	0.146	0.148	0.167	0.158	0.187	0.315	0.272	0.414
2022	0.158	0.132	0.152	0.15	0.154	0.165	0.172	0.311	0.327	0.363
Mean	0.143	0.129	0.116	0.132	0.137	0.141	0.170	0.300	0.388	0.312

The overall Gini coefficient of the CCD between two systems increased from 0.148 to 0.158 between 2015 and 2022, indicating that the CCD disparity between the two systems has widened. The largest increase in the overall Gini coefficient of the CCD occurred between 2019 and 2020. This suggests that the COVID-19 pandemic exacerbated regional disparities in the CCD.

From the perspective of within-regional differences, the average internal differences were the largest in the western region, followed by the eastern region, while the average internal differences were the smallest in the central region. From the perspective of inter-regional differences, East–West disparities consistently exceeded both East-Central differences and Central-West differences.

From the perspective of the sources of these differences, between 2015 and 2022, the average contribution rates of within-regional differences, inter-regional differences, and hypervariable density to the spatial differences in CCD for digital rural development and rural healthcare service efficiency were 29.00, 47.30, and 23.70%, respectively. Additionally, from 2015 to 2019, hypervariable density had the smallest driving effect on CCD spatial differences, while inter-regional differences dominated, followed by within-regional differences. Between 2019 and 2022, the situation has reversed, and the hypervariable density has become the main factor responsible for these spatial differences. This is because some provinces achieved rapid progress during the pandemic by relying on digital governance and remote medical systems, while other provinces lagged behind due to weak digital infrastructure. This phenomenon has made the distribution differences between different regions even more pronounced. This shows that during the pandemic, the improvement in the efficiency of digital villages and rural medical services in China was not balanced, but rather presented a pattern of rapid development coexisting with slow development. In addition, the impact of inter-regional differences first decreased and then increased, while within-regional differences showed the opposite trend. This shows that, following the COVID-19 pandemic, within-regional differences were greater than inter-regional gaps.

### 5.3 Spatio-temporal evolution characteristics of the CCD between two systems

#### 5.3.1 Temporal evolution characteristics of the CCD between two systems

**Figure 3:**
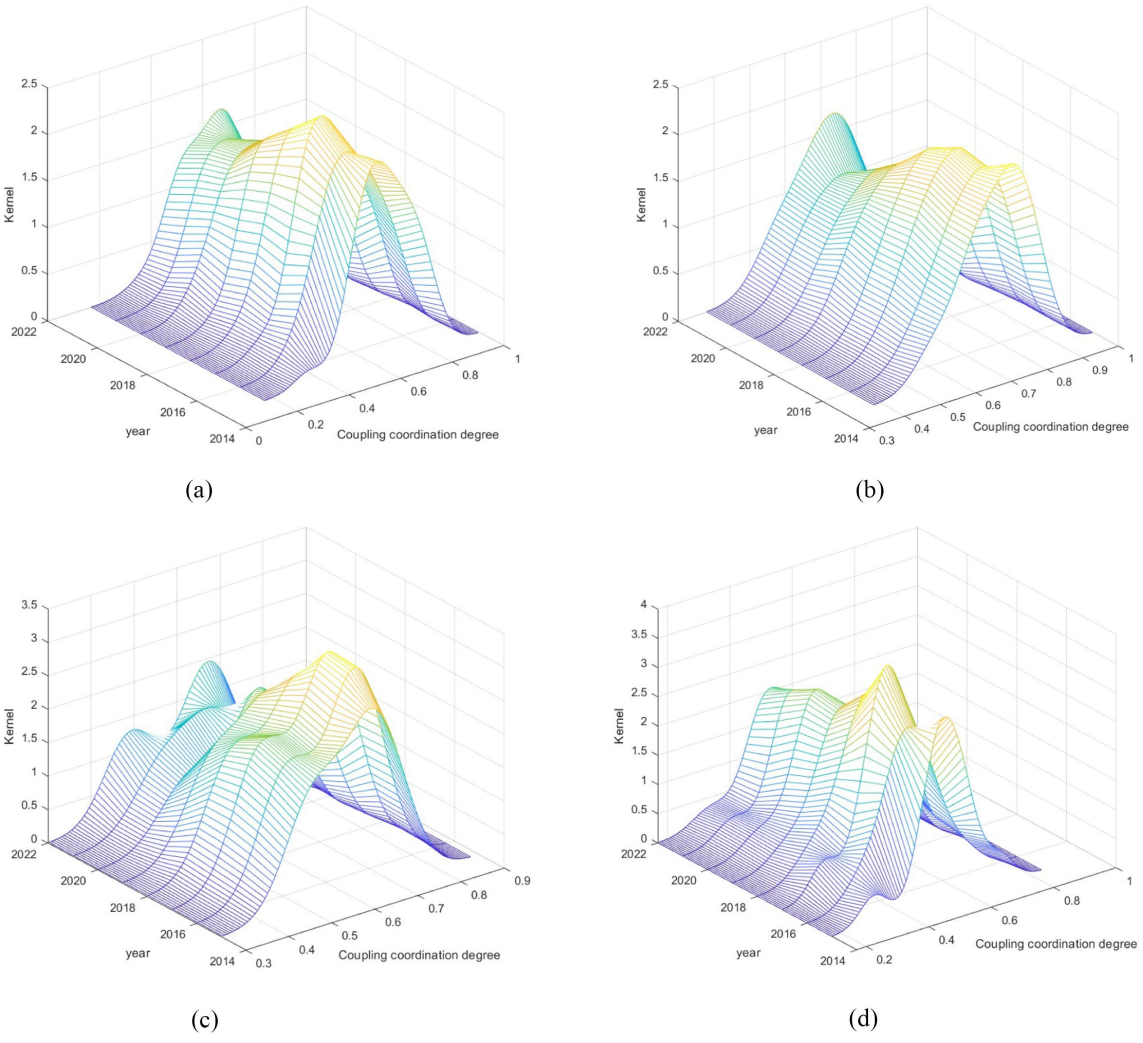
Dynamic evolution of CCD between two systems. **(a)** National, **(b)** East, **(c)** Central, **(d)** West.

To further investigate the dynamic evolution characteristics of the CCD between digital village construction and rural healthcare service efficiency, kernel density estimation was used for analysis, with the results shown in Figure 3a. From the curve distribution, the main peak position of the kernel density curve shifted to the right from 2015 to 2022, indicating that the CCD between digital village construction and rural healthcare service efficiency has been steadily improving nationwide. From the perspective of curve extensibility, the widening of the right tail of the kernel density curve in each year gradually decreases, indicating that the gap in the CCD between the two systems is narrowing from 2015 to 2022. Examining the kernel density curves over time, the diminishing spread of the right tail from 2015 to 2022 suggests a steady convergence in the CCD disparity between the two systems. The persistent unimodal distribution throughout the study period further reveals that polarization between China’s digital village development and rural healthcare efficiency is softening, with both systems progressively trending toward equilibrium.

From a regional perspective, the results of the kernel density analysis of the CCD between the two systems in each region from 2015 to 2022 are shown in Figures 3b–d. In terms of distribution location, the curve in the central region shows a trend of continuous movement to the right every year, while the eastern and western regions show a more complex trajectory, that is, first moving to the left and then turning to the right. In terms of curve extension, the kernel density map of the eastern region shows a significant expansion at the right end of the survey period, indicating that the region far exceeds the benchmark province in terms of the compatibility and effectiveness of digital rural initiatives with improving the efficiency of rural healthcare services. The kernel density curves in the central and western regions all show left-tail characteristics. The left tail in the central region is gradually shortening, indicating that the gap between provinces with low CCD levels is narrowing, while the left tail in the western region is gradually lengthening, indicating that the gap between provinces with low CCD levels is widening. In terms of peak curves, the eastern region only maintained one peak throughout the study period, which means that the region is currently relatively lightly differentiated The central area displayed a shift from a “single peak” to a “double peak,” pointing to the growing prominence of the polarization issue in that region. Then, the western area demonstrated a move from “double peaks” to “multiple peaks,” signifying that the polarization is surely getting more intense.

#### 5.3.2 Spatial evolution characteristics of the CCD between two systems

The kernel density estimation technique is limited to offering a basic depiction of how the CCD between digital village development and the effectiveness of rural healthcare services fluctuates over time. However, it lacks the capability to delve into the spatial patterns and characteristics of their distribution dynamics. The advantage of the Markov chain method lies in providing dynamic information on regional movements within the distribution (62). Therefore, this study employs conventional and spatial Markov transition probability matrices. Using a quartile classification approach, the CCD is categorized into four distinct tiers: low (k = I), medium-low (k = II), medium-high (k = III), and high (k = IV). The traditional Markov transition probability matrix is shown in Table 7.

**Table 7:** Traditional Markov transfer probability matrix for the CCD in China, 2015–2022.

Type of lag	I	II	III	IV
I	0.820	0.160	0.020	0.000
II	0.127	0.745	0.127	0.000
III	0.000	0.020	0.860	0.120
IV	0.000	0.000	0.021	0.979

Table 7 shows that the diagonal probability values (minimum probability value of 0.745) of the CCD between digital rural construction and rural healthcare service efficiency in the traditional Markov transition probability matrix are significantly higher than the off-diagonal probability values (maximum probability value of 0.160), indicating that the CCD exhibits strong stability and significant path dependence at the provincial spatial scale. Additionally, the probability values in the lower right diagonal of the CCD transition probability matrix (0.860 and 0.979) are significantly higher than those in the upper left diagonal (0.820 and 0.745), indicating that provinces with higher CCD are more likely to maintain stability, exhibiting a certain degree of club convergence. Furthermore, the transition of CCD is more active between similar levels, indicating that achieving coordinated development in digital rural construction and rural healthcare service efficiency is a gradual process that is difficult to be realised through short-term leapfrog growth.

Traditional Markov analysis treats each region as an independent unit when reflecting the transitional characteristics of the CCD between digital village construction and rural healthcare service efficiency in different regions, without considering the influence of surrounding adjacent types on their evolution (63). Therefore, this study employed a spatial adjacency weight matrix to analyze the global Moran’s I index, assessing the CCD between digital village development and rural healthcare efficiency from 2015 to 2022. The findings reveal that all *p*-values for the global Moran’s I index fall below 0.05, demonstrating a statistically significant and spatially clustered relationship between these two factors across China (The calculation results are shown in Supplementary material). Therefore, incorporating the spatial factor into the study’s scope is necessary for developing a spatial Markov transition probability model. The calculation results are shown in Table 8.

**Table 8:** Spatial Markov transfer probability matrix for the CCD in China, 2015–2022.

Period lag type	Spatial lag type	Target area type	I	II	III	IV
t/t + 1	I	I	0.800	0.200	0.000	0.000
		II	0.111	0.889	0.000	0.000
		III	0.000	0.000	0.000	0.000
		IV	0.000	0.000	0.000	1.000
	II	I	0.815	0.185	0.000	0.000
		II	0.154	0.769	0.077	0.000
		III	0.000	0.000	1.000	0.000
		IV	0.000	0.000	0.000	1.000
	III	I	0.667	0.167	0.167	0.000
		II	0.111	0.722	0.167	0.000
		III	0.000	0.000	0.800	0.200
		IV	0.000	0.000	0.037	0.963
	IV	I	1.000	0.000	0.000	0.000
		II	0.000	0.000	1.000	0.000
		III	0.000	0.056	0.889	0.056
		IV	0.000	0.000	0.000	1.000

Table 8 shows that, compared with the traditional Markov transition probability matrix that does not consider spatial effects, the CCD between digital village construction and rural healthcare service efficiency exhibits different transition characteristics under different neighbourhood contexts. For provinces with a low CCD, when they are adjacent to provinces with a high CCD, the probability of upward transition increases. After considering spatial factors, the probability values on the diagonal of the spatial Markov transition probability matrix are generally higher than those off the diagonal, further confirming the club convergence phenomenon in the spatial dimension. Compared to a 1 year lag, under 2 years and 3 years lag conditions (The calculation results are shown in Supplementary material), the probability of upward transition increases over time when provinces with lower levels are adjacent. When medium-low-level provinces are adjacent to medium-high-level provinces, the probability of medium-high-level provinces moving upward increases over time, while the probability of medium-low-level provinces moving downward increases, indicating that medium-high-level regions exert a siphoning effect on medium-low-level regions; when high-level provinces are adjacent to medium-high-level provinces, the probability of medium-high-level provinces moving upward increases over time.

### 5.4 Analysis of the influencing factors of the CCD between two systems

Table 9 presents the results of quantile regression analysis. LED, GOV, HDI, and DIN all have a significant positive impact on CCD. Specifically, the β coefficients for LED in the 0.4 to 0.8 quantile fluctuate between 0.118 and 0.169, showing a trend of first increasing and then decreasing. All coefficients pass the 1% significance test. This indicates that economic development levels have a positive effect on CCD, with higher economic development levels leading to higher CCD. HNL have a positive impact on CCD. In the 0.2–0.9 quantile, the trend is generally increasing. The β coefficients range from 0.062 to 0.225. DIN has a significant positive impact on CCD at the 0.4–0.9 quantile, with β coefficients ranging from 0.041 to 0.111, all passing the significance test at the 5% level, indicating that as digital innovation capacity improves, CCD also increases accordingly. GOV has a significant positive impact on CCD at the 0.1–0.8 quantile, with its β coefficient fluctuating between 0.117 and 0.139, indicating that increases in government health expenditure can promote an increase in CCD. Additionally, the results indicate that HHC and URB have a negative impact on CCD. Specifically, HHC has a significant negative impact on CCD at the 0.1–0.9 quantile, with its β coefficient fluctuating between −0.110 and −0.275 and showing a decreasing trend. While URB only have a significant positive impact on CCD at the 0.4–0.8 quantile, with the β coefficient fluctuating between −0.239 and −0.372, showing an initial increase followed by a decrease.

**Table 9:** Regression analysis of quantile results of influencing factors.

Index	10%	20%	30%	40%	50%	60%	70%	80%	90%
LED	0.000	0.068	0.047	0.118^***^	0.157^***^	0.159^***^	0.169^***^	0.155^***^	−0.003
	(0.008)	(1.522)	(1.165)	(3.721)	(6.078)	(7.323)	(8.922)	(7.286)	(−0.088)
HNL	0.046	0.062^*^	0.062^**^	0.069^***^	0.081^***^	0.097^***^	0.090^***^	0.106^***^	0.225^***^
	(0.918)	(1.816)	(2.093)	(2.664)	(3.548)	(4.797)	(4.742)	(5.000)	(8.517)
DIN	−0.030	0.014	0.034	0.053^**^	0.063^***^	0.041^**^	0.033	0.036	0.111^***^
	(−0.747)	(0.469)	(1.163)	(2.069)	(2.806)	(2.049)	(1.834)	(1.796)	(3.767)
GOV	0.116^***^	0.133^***^	0.139^***^	0.118^***^	0.116^***^	0.123^***^	0.129^***^	0.117^***^	0.005
	(3.095)	(4.168)	(5.086)	(5.063)	(5.841)	(7.228)	(8.593)	(7.197)	(0.187)
HHC	−0.275^***^	−0.222^***^	−0.178^***^	−0.164^***^	−0.155^***^	−0.151^***^	−0.153^***^	−0.110^***^	−0.047^*^
	(−10.113)	(−7.641)	(−7.228)	(−7.089)	(−6.861)	(−6.793)	(−6.567)	(−3.749)	(−1.738)
URB	0.160	−0.030	−0.133	−0.263^***^	−0.372^***^	−0.323^***^	−0.281^***^	−0.239^***^	−0.143
	(0.972)	(−0.242)	(−1.137)	(−2.626)	(−4.285)	(−4.249)	(−4.119)	(−3.080)	(−1.239)
Constant	−2.848^**^	−3.279^***^	−2.789^***^	−2.495^***^	−2.498^***^	−3.053^***^	−3.423^***^	−3.309^***^	0.079
	(−2.358)	(−3.170)	(−2.888)	(−2.992)	(−3.525)	(−5.071)	(−6.581)	(−5.840)	(0.087)
Observations	232	232	232	232	232	232	232	232	232

The symbols *, **, *** denote significance levels of 10, 5, and 1%, respectively.

## 6 Discussion

### 6.1 Coupling coordination degree improves steadily but requires further strengthening

This study indicates that between 2015 and 2022, the CCD between the level of digital village construction and the efficiency of rural healthcare services showed a steady upward trend, with the overall CCD level still in the initial coordination stage. This conclusion is similar to existing research (30). This suggests that policies aimed at developing rural digital infrastructure and enhancing rural healthcare service capabilities have achieved remarkable results. However, some provinces remain in a transitional or imbalanced stage, and need further improvement to achieve comprehensive coordination. Additionally, the eastern region exhibits a higher level of CCD than the central and western regions. This is consistent with existing research findings (29), mainly due to its higher level of economic development, developed digital infrastructure and abundant medical resources (18). Furthermore, the level of CCD in central and western regions have been steadily improving, which may be attributed to government policies aimed at narrowing regional development gaps, such as the Western Development Strategy and the Central Region Rise Strategy, as well as poverty alleviation measures and rural healthcare support policies for poor counties in the central and western regions (64). During the period from 2019 to 2021, the national CCD level experienced a brief decline. This fluctuation is consistent with the outbreak of the COVID-19 epidemic, which appears to have acted as an important external factor potentially disrupting the coordinated evolution of the two systems. During the epidemic, resources were prioritized for epidemic prevention and control work, which likely led to the suspension of some digital projects and the slowdown in rural infrastructure construction (38), thereby contributing to a decline in the synergistic efficiency between digital villages and healthcare systems. However, as the pandemic eased and relevant policies continued to be implemented, the CCD gradually recovered and rebounded starting from 2022, reflecting the system’s apparent resilience and adaptability. The COVID-19 pandemic underscored digital innovation’s critical impact on health crises. Digitizing primary healthcare may help narrow rural disparities and foster equitable access, advancing global digital health goals.

### 6.2 Interregional gaps narrow while intra-regional differentiation intensifies

We calculated Dagum’s Gini coefficient and found that before 2019, differences in China’s CCD were mainly reflected in development gaps between regions. This may be attributed to the fact that eastern regions implemented digital health strategies earlier, were the first to adopt digital technologies, and had abundant resources, while central and western regions had insufficient digital infrastructure and weak rural healthcare systems, and had not yet fully realised the benefits of policy measures (65). After 2019, the disparities gradually shifted from inter-regional to within-regional. This may be explained by the construction of digital villages and the efficiency of rural healthcare services have been affected by the external impact of the COVID-19 epidemic, potentially leading to an unbalanced allocation of medical resources within the region, which in turn has intensified resource competition within the region. The peak pattern of the kernel density curve in the central and western regions has shifted to “double peaks” or “multiple peaks,” This conclusion is similar to that reached in some studies (66), which indicates that some provinces in the region have development gaps and gradient differences in the level of coordinated development. Similar regional disparities have been observed in international rural digital health initiatives. India’s experience demonstrates that the integration of digital technologies with primary healthcare yields markedly different outcomes across regions due to factors including socio-political contexts. This underscores the necessity for digital health strategies to be localised and dynamically adapted (67). Moreover, this phenomenon may also be linked to the siphoning effect between regions and the uneven distribution of resources within regions. As national policies increasingly tilt towards the central and western regions, digital health resources and medical insurance systems in these regions are gradually improving. Some economically developed provinces have played a synergistic effect, giving priority to promoting the transformation and upgrading of economic structures, and a large amount of digital health resources have been effectively allocated to these provinces. Although this synergistic effect promotes regional development, the siphon effect, which is stronger than the spatial spillover effect, may aggravate internal differences and lead to structural differentiation in which the strong and the weak coexist. Currently, reducing regional health disparities is not only a crucial objective for a Healthy China but also a key issue in the World Health Organization’s Sustainable Development Goals.

### 6.3 Dual characteristics of interregional synergistic development and siphoning effect

The results of the spatial Markov chain analysis indicate that provinces with higher levels of coordinated development in digital village construction and rural healthcare service efficiency can play a certain role in promoting coordinated development in surrounding provinces. Additionally, there is a noticeable synergy effect between regions with lower CCD levels and those with higher CCD levels. This result is similar to existing research (27). High-level regions have established relatively well-developed digital infrastructure and healthcare systems, with smaller development gaps, leading to resource flows and policy interactions that exhibit multi-dimensional and balanced characteristics. They are more likely to achieve regional overall improvement and coordinated development through healthy competition and cooperation. Additionally, higher-level provinces are geographically adjacent, which facilitates smoother cooperation and information exchange, further reinforcing the overall advantages of regional coordination. Low-level regions face similar development challenges. On the one hand, they tend to break through bottlenecks through resource sharing and technological cooperation. On the other hand, the long-term policy support has driven the development of low-level regions. It is worth noting that the study found that some medium-to-high-level regions exhibit a certain siphon effect on surrounding medium-to-low-level regions. This is a new discovery. This phenomenon may stem from the significant asymmetry in resource competition between medium-to-high-level regions and medium-to-low-level regions. Medium-to-high-level regions are currently breaking through the stage of economies of scale, characterised by high marginal returns and strong resource attractiveness. In addition, its governance capabilities and policy implementation efficiency advantages are obvious, making it easier for it to absorb surrounding resources unilaterally.

### 6.4 The development of CCD has been influenced by multiple factors

The results of the quantile regression method indicate that economic development has a positive incentive effect on CCD in the middle and high quantiles, which is consistent with existing research (59). The reason may be that higher levels of economic development can bring better fiscal revenue and industrial support, thereby promoting investment in digital infrastructure and medical resources and improving CCD. This is evident in lower and middle income regions like Bangladesh, where economic barriers hinder the expanded impact of digital primary healthcare (68). At the median level, the marginal benefits brought about by economic development are greater, and the driving force is obvious. However, at the high level, more investment is directed towards high-end industries or urban construction, and the marginal benefits of rural healthcare and digital construction decline, which may weaken the positive effect.

Closely related to this, the impact of residents’ health needs also varies across quantiles. In areas with low resource endowments, even though residents exhibit strong health demand, the shortage of supply prevents effective responses, potentially leading to a decline in CCD. By contrast, in higher quantiles where digital and medical systems are more established, demand can be quickly met, forming a virtuous cycle among demand, supply, and policy.

A similar pattern can be observed in digital innovation. Digital innovation capabilities only have a significant positive effect in high quartiles, which is consistent with existing research findings (30). This may be due to the fact that digital innovation requires strong scientific research capabilities, industrial chain support, and a favourable financial environment, all of which are concentrated in economically developed regions. In lower ranked regions, innovation outcomes lack conversion platforms and application scenarios, making it difficult to improve the level of coordinated development.

By contrast, government health expenditure plays a compensatory role on CCD in low quartiles, which is consistent with existing research (29). This is because in regions with low CCD levels, due to insufficient market mechanisms, government fiscal expenditure has become a key incremental resource that can directly improve grassroots infrastructure and promote the implementation of digitalisation projects.

Health human capital has a significant negative impact at the 0.1–0.9 percentile. This result indicates that simply increasing the number of rural primary healthcare personnel has not effectively improved CCD, which differs from previous studies. One possible reason is that the current structure of rural healthcare personnel is unreasonable, with a focus on quantity over quality, lacking digital skills and multidisciplinary talent. Another possible reason is that rural areas lack digital equipment and information systems, preventing healthcare personnel from functioning efficiently and leading to personnel redundancy. In addition, the lack of incentive mechanisms is also a key factor. The existing human resource allocation mechanism may cause high-quality medical personnel to continue to move to cities, while those remaining in rural areas lack motivation, thereby affecting service quality. At the same time, the lagging training system has exacerbated this problem. The existing digital skills training system is still imperfect and unable to meet the actual needs of digital medical services, leading to a disconnect between human resources and technological development.

Finally, urbanisation rates have a significant negative effect in the middle and high quartiles, which contradicts existing research findings (59). As the population concentrates in cities, the size and demand of the rural population shrinks. The siphoning effect brought about by rapid urbanisation may have led to a further loss of medical and digital resources in rural areas (66). At the same time, the widening urban–rural gap has reduced the investment appeal of digital healthcare in rural areas, exacerbating systemic imbalances.

### 6.5 Policy implications for promoting balanced rural digital health development

Based on China’s current experience, in order to achieve comprehensive and coordinated development, we must continue to strengthen regional differentiated development strategies and accurately implement policies that are in line with the development foundations of different regions. It is necessary to give full play to the leading role of the eastern region and build a cross-provincial technology and resource sharing cooperation platform. This initiative aims to integrate healthcare resources, reduce costs, and enable residents in less developed areas to access premium remote medical services more extensively. This is not only conducive to the exchange of experience and promotes digital infrastructure construction and talent training in the central and western regions, but also provides a valuable example for other developing countries seeking to promote digital rural development and improve medical capabilities through regional collaboration.

In view of the continued widening regional gap, it is necessary to establish a medical resource decentralization mechanism within the province, encourage tertiary hospitals to establish long-term and stable assistance cooperation mechanisms with county-level hospitals and grassroots medical and health institutions, and promote the radiation of medical resources to surrounding towns and villages (69), thereby alleviating regional gaps and ensuring that high-quality medical resources effectively benefit grassroots communities. Similarly, the government must enhance financial support for the interoperability of electronic health records across townships and villages to improve the sharing of primary healthcare information and reduce the fragmentation of medical services. This will ensure that the benefits of digitalisation genuinely reach the grassroots population.

Faced with the siphon effect of neighboring provinces, China and other developing countries should improve regional development assessment mechanisms when promoting digital health policies to prevent excessive concentration of high-quality resources and policy funds in high-level areas. Such reforms can not only promote balanced development among regions, but also provide a theoretical basis for other countries to promote the development of rural digital healthcare.

Given the numerous factors influencing the development of CCD, China should adopt policies that redirect the benefits of economic growth toward rural areas, promote the dissemination of digital innovation outcomes, and optimise fiscal investments. This will strengthen the positive feedback loop between demand and supply, while improving the configuration of basic infrastructure and enhancing the coverage and payment functions of the medical insurance system in rural areas to enhance residents’ ability to pay. Additionally, efforts should be made to improve the structure and digital skills of rural healthcare personnel, avoiding excessive expansion that leads to surplus staff, while establishing incentive mechanisms to attract high-quality healthcare talent to settle in rural areas. This will promote the balanced integration of rural digitalisation and healthcare services for high-quality development.

### 6.6 Limitations and future research

Due to data constraints, this research does not cover the digital countryside or the effectiveness of rural health services in Beijing, Shanghai, Hong Kong, Macao, and Taiwan between 2015 and 2022. It should be clarified that although Beijing and Shanghai lead in digitalisation and healthcare provision, their rural populations are relatively small and both are located in eastern China, thus their exclusion has a limited impact on the study’s conclusions. Furthermore, China lacks a fully developed and methodologically sound comprehensive assessment framework for digital village initiatives and rural healthcare efficiency, which may lead to some shortcomings in how the indicators were chosen. Furthermore, the CRITIC method employed in this study is an objective weighting method, which to some extent lacks subjective judgement regarding the importance of each indicator. The available statistical resources on rural digital development and healthcare infrastructure are limited, restricting the scope of this study to provincial-level analysis within China.

In view of the above deficiencies, future research should further strengthen data collection and integration, and at the same time adopt a combination of subjective and objective empowerment methods to gradually build a multi-dimensional indicator system covering the whole country and reaching down to the county level, so as to enhance the explanatory power of data. and applicability of conclusions. Further research should also be carried out on the key influencing factors that promote the coupling and coordination of digital villages and rural medical services, so as to provide a basis for building a long-term sustainable digital rural health development path.

## 7 Conclusion

This study employed a coupled coordination degree model to assess the level of coupled coordination between digital rural development and rural healthcare service efficiency in 29 provinces (including municipalities and autonomous regions) across China. This study utilized methods such as Dagum’s Gini coefficient, kernel density estimation, spatial Markov chains, and quantile regression to explore regional differences, spatiotemporal evolution characteristics, and driving factors of the coupling and coordinated development between digital rural development and rural healthcare service efficiency. The study reached the following key conclusions:

(1) The overall level of digital village construction across China’s provinces has improved, and the efficiency of rural healthcare services has gradually recovered post-pandemic. Among these, the eastern regions outperform central and western regions in both digital village construction and rural healthcare service efficiency, showing a gradual decline from east to west.
(2) The level of CCD between digital rural construction and rural healthcare service efficiency continues to improve, and is currently in the primary coordination stage. Regional differences are very significant. The eastern region is in a leading position, the central region is facing polarization problems, and the western region is showing an increasing trend of multipolarity.
(3) The CCD of digital rural construction and rural medical service efficiency between adjacent provinces has significant spatial spillover effects, and high-coordination areas play a leading role in low-coordination areas. As time progresses, regions with high CCD levels exhibit a clear mutually reinforcing relationship with regions with low CCD levels, while regions with moderate CCD levels may experience a siphoning effect. Regional CCD and development still require further strengthening.
(4) Economic development, health needs, digital innovation, and government investment are key drivers for improving the CCD, but neglecting to optimise human resource structures and balance urban and rural medical resources may weaken the CCD level.

## Data Availability

The datasets presented in this study can be found in online repositories. The names of the repository/repositories and accession number(s) can be found in the article/Supplementary material.
