# Supplementary material for: The coupling coordination between digital village construction and rural healthcare service efficiency in China: dynamic evolution, spatial difference and driving factors
Source: Front Public Health. 2025 Oct 13;13:1669695. doi: 10.3389/fpubh.2025.1669695 (PMC12554649; doi:10.3389/fpubh.2025.1669695)
Supplement: Supplementary file 1 [file Table_1.DOCX]

Supplementary Material

# Supplementary Tables

## The results of global Moran’s I value are shown in **Supplementary** Table 1.

**Supplementary Table 1**　Global Moran’ I index variation of the CCD between the two systems from 2015 to 2022

| **Year** | **I value** | **Z value** | **P value** |
| --- | --- | --- | --- |
| 2015 | 0.280 | 2.606 | 0.005^***^ |
| 2016 | 0.277 | 2.535 | 0.006^***^ |
| 2017 | 0.284 | 2.604 | 0.005^***^ |
| 2018 | 0.352 | 3.150 | 0.001^***^ |
| 2019 | 0.337 | 3.028 | 0.001^***^ |
| 2020 | 0.360 | 3.207 | 0.001^***^ |
| 2021 | 0.348 | 3.113 | 0.001^***^ |
| 2022 | 0.407 | 3.603 | 0.000^***^ |

Note: The symbols *, **, *** denote significance levels of 10%, 5%, and 1%, respectively.

## The spatial Markov transition probability matrix under two year and three year lag conditions is shown in **Supplementary Supplementary** Table 2.

**Supplementary Table 2**　Spatial Markov transfer probability matrix for the CCD in China, 2015–2022.

| **Period lag type** | **Spatial lag type** | **Target area type** | **I** | **II** | **III** | **IV** |
| --- | --- | --- | --- | --- | --- | --- |
| t / t+2 | I | I | 0.667 | 0.333 | 0.000 | 0.000 |
|  |  | II | 0.125 | 0.875 | 0.000 | 0.000 |
|  |  | III | 0.000 | 0.000 | 0.000 | 0.000 |
|  |  | IV | 0.000 | 0.000 | 0.000 | 1.000 |
|  | II | I | 0.708 | 0.292 | 0.000 | 0.000 |
|  |  | II | 0.333 | 0.542 | 0.125 | 0.000 |
|  |  | III | 0.000 | 0.000 | 0.800 | 0.200 |
|  |  | IV | 0.000 | 0.000 | 0.000 | 1.000 |
|  | III | I | 0.250 | 0.500 | 0.250 | 0.000 |
|  |  | II | 0.235 | 0.588 | 0.176 | 0.000 |
|  |  | III | 0.000 | 0.000 | 0.739 | 0.261 |
|  |  | IV | 0.000 | 0.000 | 0.045 | 0.955 |
|  | IV | I | 1.000 | 0.000 | 0.000 | 0.000 |
|  |  | II | 0.000 | 0.000 | 1.000 | 0.000 |
|  |  | III | 0.000 | 0.067 | 0.867 | 0.067 |
|  |  | IV | 0.000 | 0.000 | 0.000 | 1.000 |
| t / t+3 | I | I | 0.333 | 0.667 | 0.000 | 0.000 |
|  |  | II | 0.143 | 0.857 | 0.000 | 0.000 |
|  |  | III | 0.000 | 0.000 | 0.000 | 0.000 |
|  |  | IV | 0.000 | 0.000 | 0.000 | 1.000 |
|  | II | I | 0.810 | 0.190 | 0.000 | 0.000 |
|  |  | II | 0.409 | 0.409 | 0.182 | 0.000 |
|  |  | III | 0.000 | 0.000 | 1.000 | 0.000 |
|  |  | IV | 0.000 | 0.000 | 0.000 | 1.000 |
|  | III | I | 0.000 | 1.000 | 0.000 | 0.000 |
|  |  | II | 0.267 | 0.533 | 0.200 | 0.000 |
|  |  | III | 0.071 | 0.000 | 0.286 | 0.643 |
|  |  | IV | 0.000 | 0.000 | 0.000 | 1.000 |
|  | IV | I | 1.000 | 0.000 | 0.000 | 0.000 |
|  |  | II | 0.000 | 0.000 | 1.000 | 0.000 |
|  |  | III | 0.000 | 0.000 | 0.800 | 0.200 |
|  |  | IV | 0.000 | 0.000 | 0.000 | 1.000 |
