# Supplementary figures and images for: The coupling coordination between digital village construction and rural healthcare service efficiency in China: dynamic evolution, spatial difference and driving factors
Source: Front Public Health. 2025 Oct 13;13:1669695. doi: 10.3389/fpubh.2025.1669695 (PMC12554649; doi:10.3389/fpubh.2025.1669695)

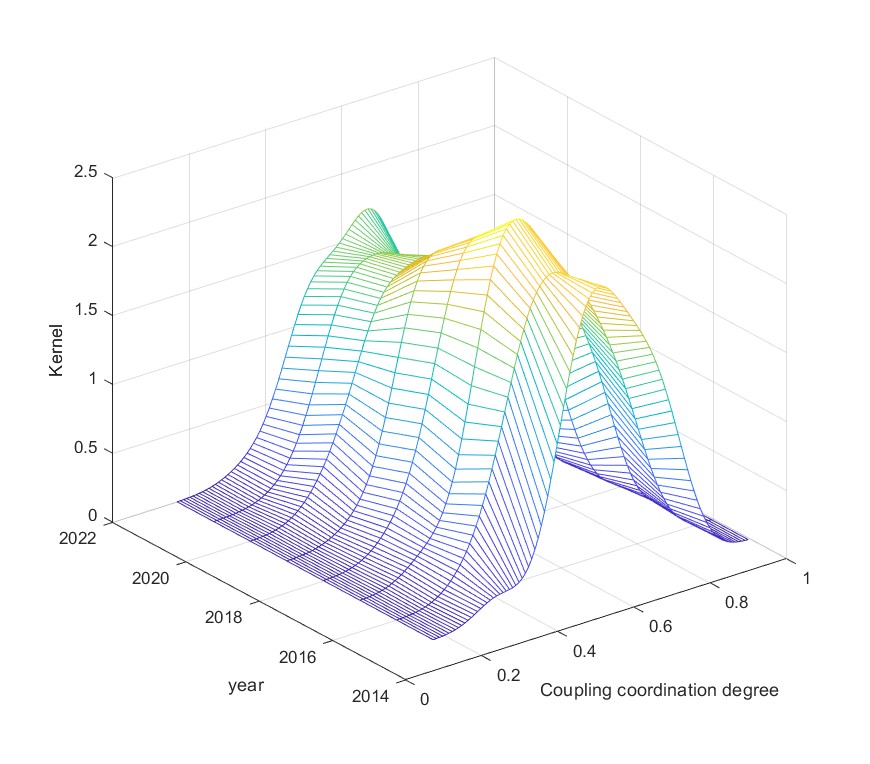

Supplement: Supplementary file 3 [file Data_Sheet_1.ZIP › Figure2 (a).National.jpg]

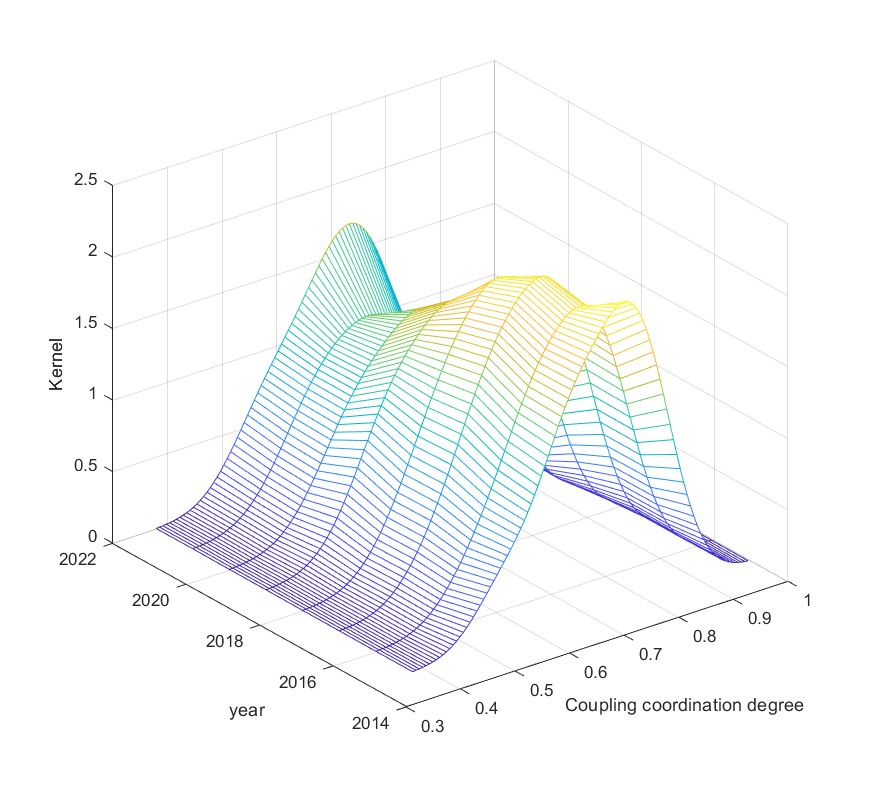

Supplement: Supplementary file 3 [file Data_Sheet_1.ZIP › Figure2 (b).East.jpg]

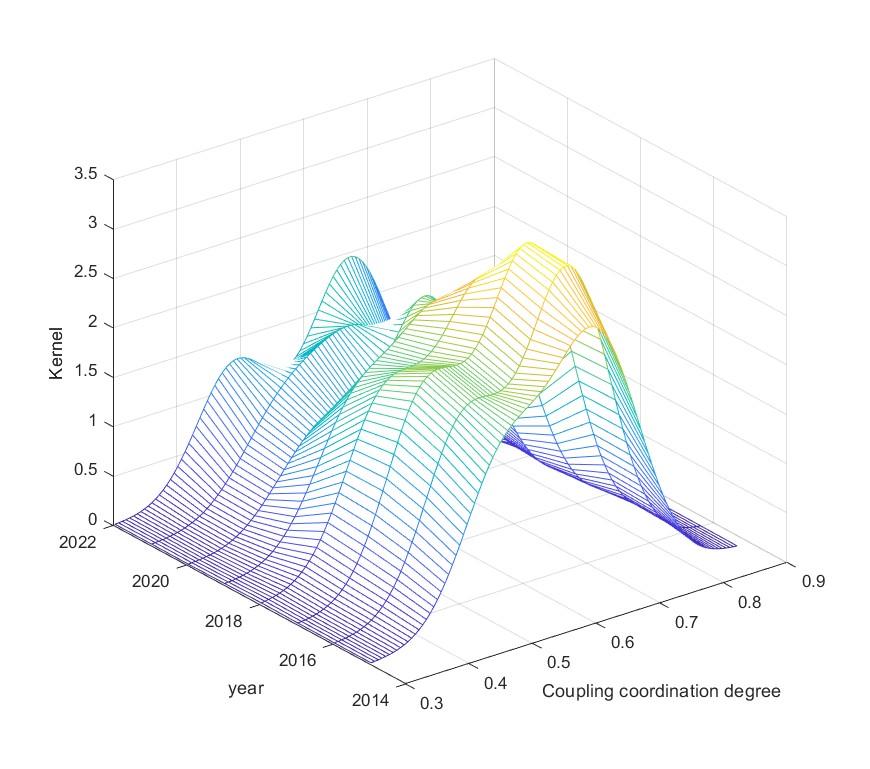

Supplement: Supplementary file 3 [file Data_Sheet_1.ZIP › Figure2 (c).Central.jpg]

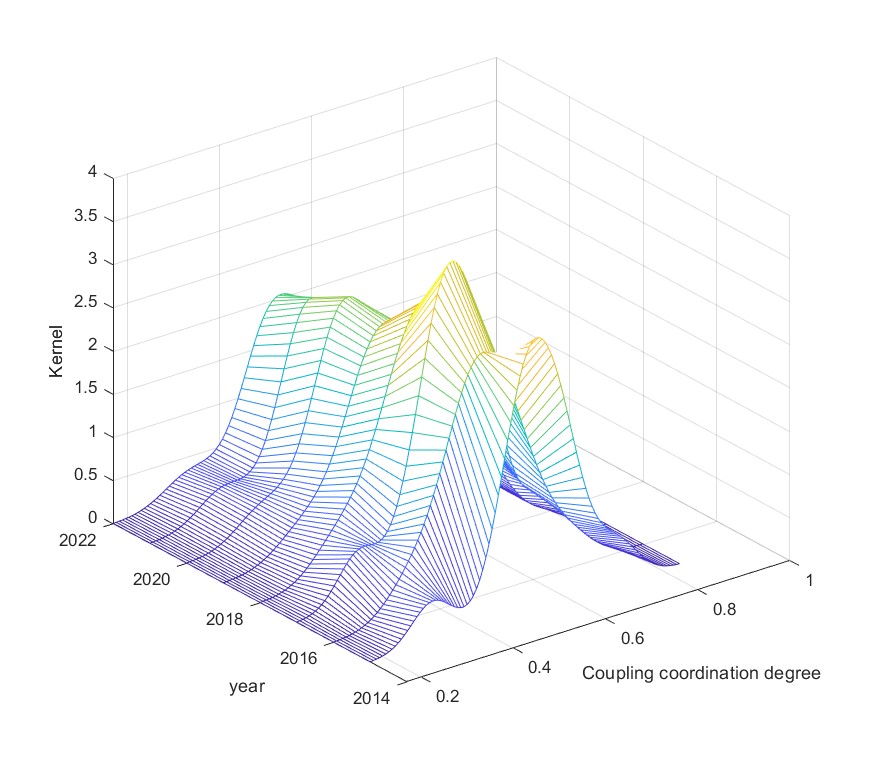

Supplement: Supplementary file 3 [file Data_Sheet_1.ZIP › Figure2 (d).West.jpg]

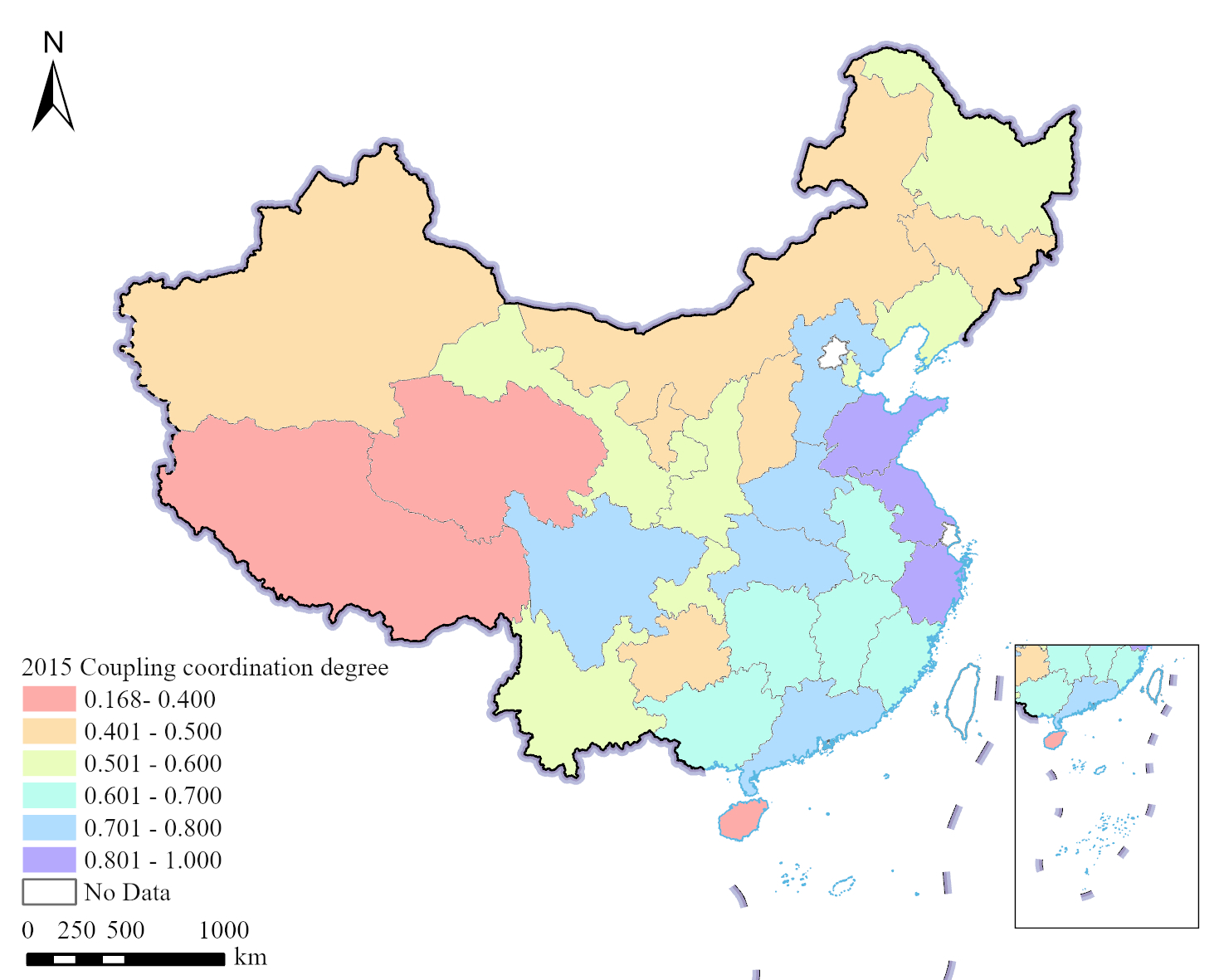

Supplement: Supplementary file 3 [file Data_Sheet_1.ZIP › Figure1 (a).2015 .jpg]

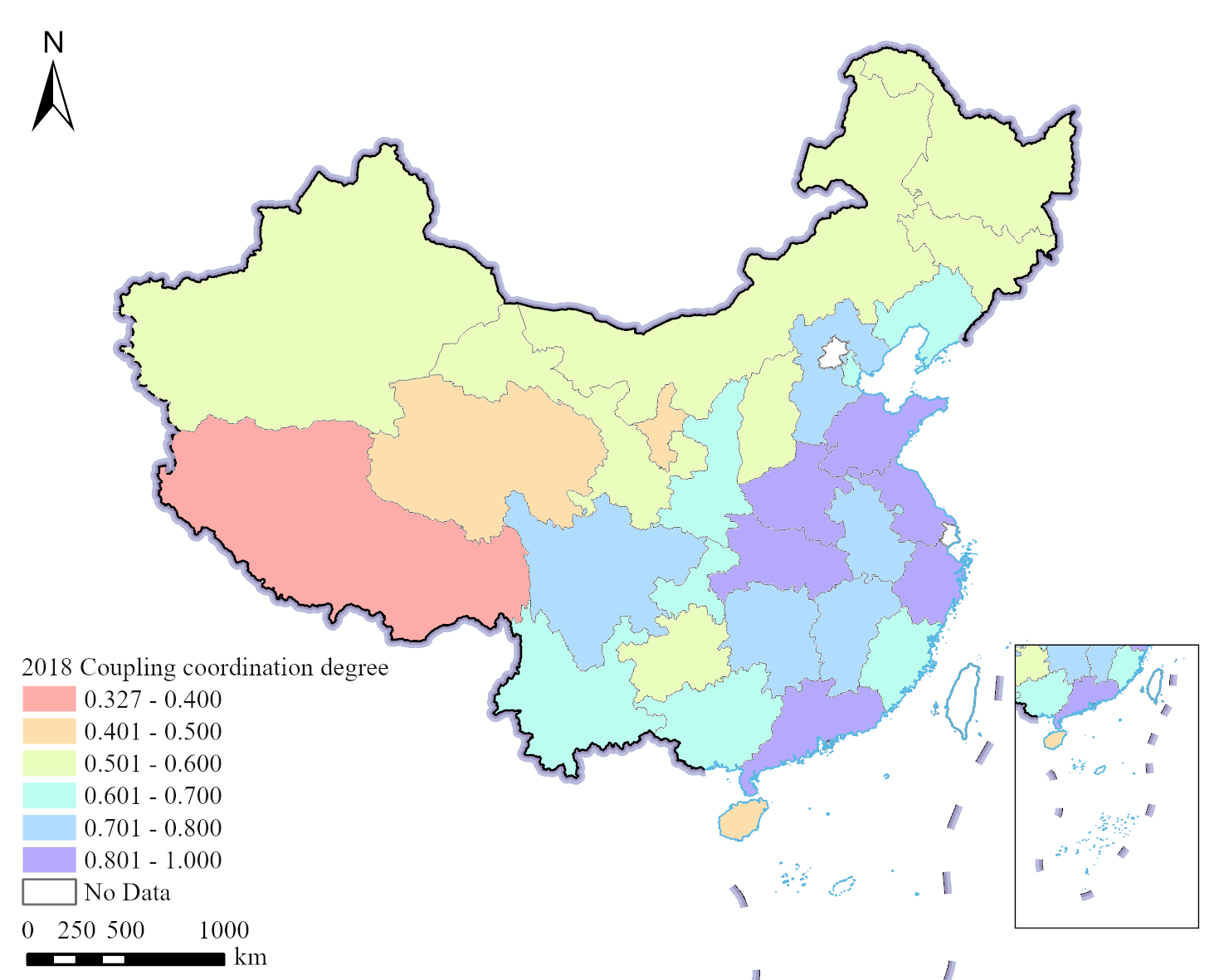

Supplement: Supplementary file 3 [file Data_Sheet_1.ZIP › Figure1 (b).2018.jpg]

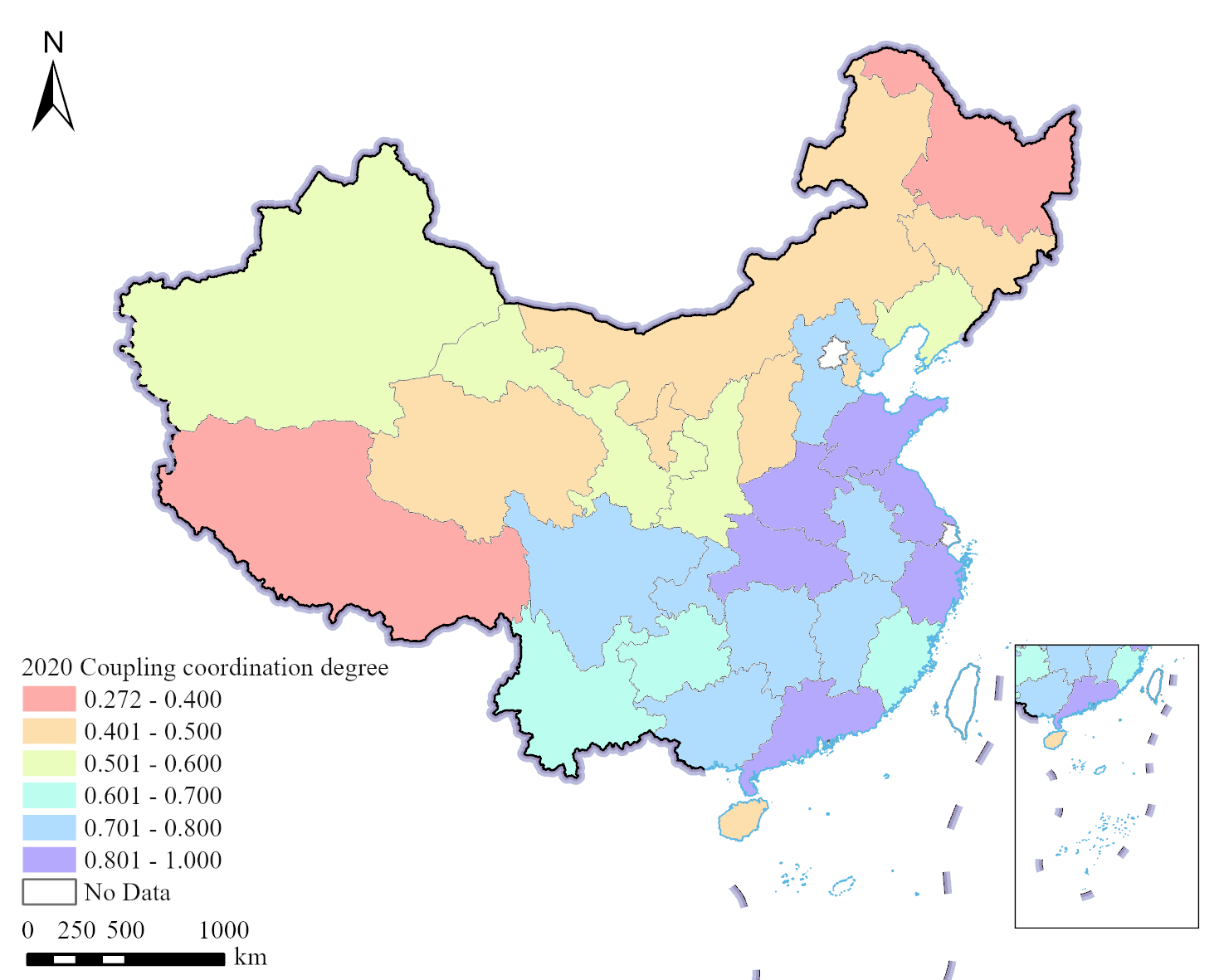

Supplement: Supplementary file 3 [file Data_Sheet_1.ZIP › Figure1 (c).2020.jpg]

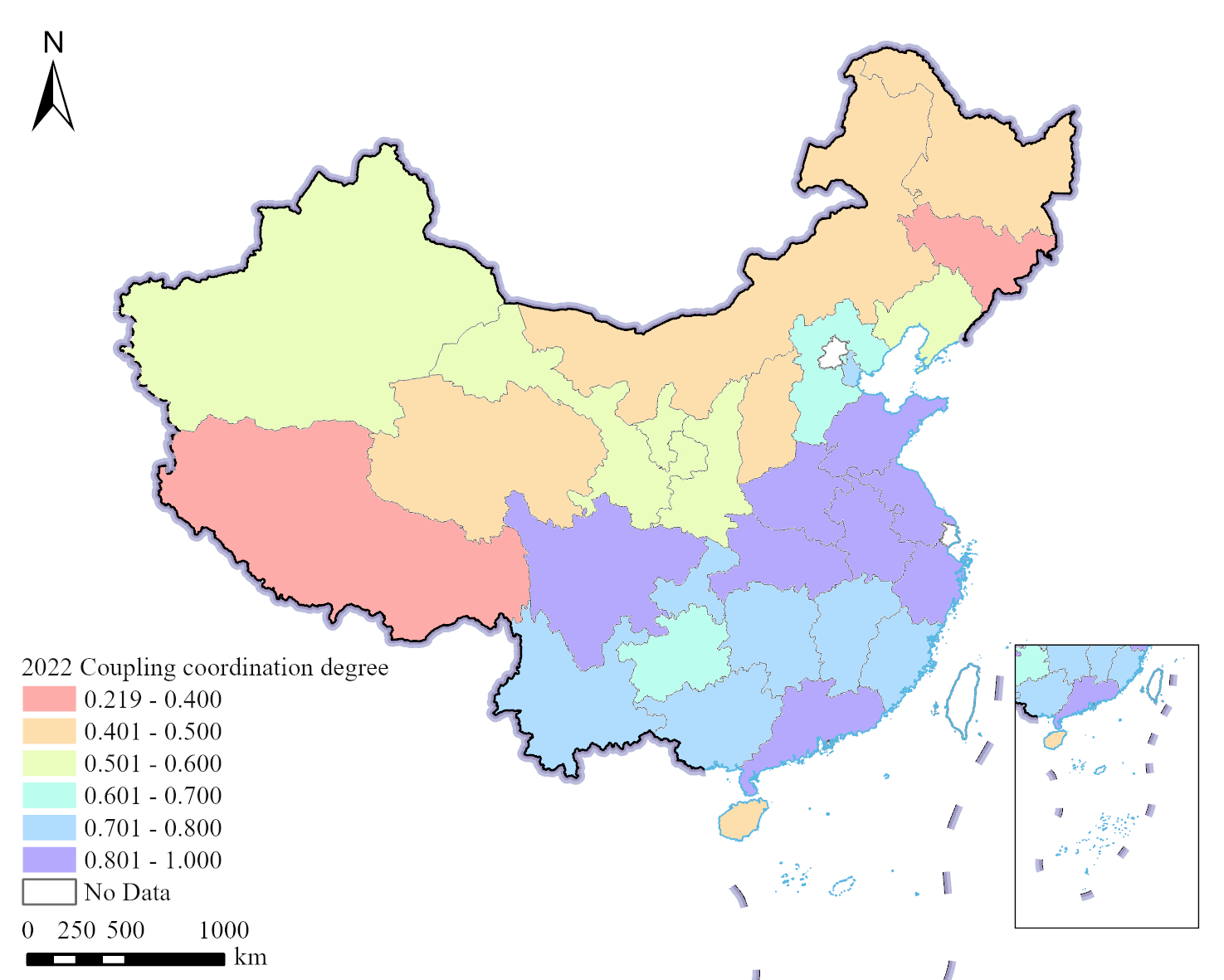

Supplement: Supplementary file 3 [file Data_Sheet_1.ZIP › Figure1 (d).2022.jpg]
